# Community health worker knowledge and management of pre-eclampsia in rural Karnataka State, India

**DOI:** 10.1186/s12978-016-0219-8

**Published:** 2016-09-30

**Authors:** Umesh Ramadurg, Marianne Vidler, Umesh Charanthimath, Geetanjali Katageri, Mrutyunjaya Bellad, Ashalata Mallapur, Shivaprasad Goudar, Shashidhar Bannale, Chandrashekhar Karadiguddi, Diane Sawchuck, Rahat Qureshi, Peter von Dadelszen, Richard Derman, the Community Level Interventions for Pre-eclampsia (CLIP) India Feasibility Working Group

**Affiliations:** 1 Department of Community Medicine, S Nijalingappa Medical College, Bagalkot, Navanagar 587102 India; 2 Department of Obstetrics and Gynaecology, and the Child and Family Research Unit, University of British Columbia, Vancouver, V5Z 4H4 Canada; 3 KLE University’s Jawaharlal Nehru Medical College, Neharu Nagar, Belgaum, 590010 India; 4 Department of Obstetrics and Gynaecology, S Nijalingappa Medical College, Bagalkot, Navanagar 587102 India; 5 Department of Pharmacology, S Nijalingappa Medical College, Bagalkot, Navanagar 587102 India; 6 Department of Research, Vancouver Island Health Authority, Victoria, V8R 1J8 Canada; 7 Division of Women and Child Health, Aga Khan University, Karachi, Pakistan; 8 Molecularand Clinical Sciences Research Institute, St George’s, University of London, and Department of Obstetrics and Gynaecology, St George’s University Hospitals NHS Foundation Trust, London, SW17 0RE UK; 9 Department of Obstetrics and Gynaecology, Thomas Jefferson University, Philadelphia, 19107 USA

**Keywords:** Blood pressure, Community health workers, Community health services, Developing countries, Eclampsia, Hypertension, India, Magnesium sulfate, Maternal health, Maternal mortality, Nurse midwives, Pre-eclampsia, Pregnancy, Proteinuria, Public health, Referral and consultation, Rural health

## Abstract

**Background:**

In India, the hypertensive disorders of pregnancy and postpartum haemorrhage are responsible for nearly 40 % of all maternal deaths. Most of these deaths occur in primary health settings which frequently lack essential equipment and medication, are understaffed, and have limited or no access to specialist care. Community health care workers are regarded as essential providers of basic maternity care; and the quality of care they provide is dependent on the level of knowledge and skills they possess. However, there is limited research regarding their ability to manage pregnancy complications. This study aims to describe the current state of knowledge regarding pre-eclampsia and eclampsia among community health care workers (auxiliary nurse midwives, accredited social health activists, staff nurses) in northern Karnataka, India. Furthermore, this study describes the treatment approaches used by various cadres of community health workers for these conditions. The findings of this study can help plan focussed training sessions to build upon their strengths and to address the identified gaps.

**Methods:**

Data were collected as part of a larger study aimed at assessing the feasibility of community-based treatment for pre-eclampsia. Eight focus group discussions were conducted in 2012–2013 in northern Karnataka State: four with staff nurses and auxiliary nurse midwives and four with accredited social health activists. In addition, twelve auxiliary nurse midwives and staff nurses completed questionnaires to explore their competence and self-efficacy in managing pre-eclampsia. Qualitative data were audio-recorded, transcribed verbatim and translated for thematic analysis using NVivo 10.

**Results:**

Community health workers described their understanding of the origins of hypertension and seizures in pregnancy. Psychological explanations of hypertension were most commonly reported: stress, tension, and fear. The most common explanation for eclampsia was not receiving a tetanus vaccination. Despite some common misperceptions regarding aetiology, these community health workers demonstrated a good grasp of the potential consequences of hypertension in pregnancy. According to auxiliary nurse midwives and staff nurses, if hypertension was detected they encouraged rest, decreased salt intake, iron supplementation and tetanus vaccination. In addition, some staff nurses administered antihypertensives, MgSO_4_, or other anticonvulsants. All auxiliary nurse midwives had some awareness of MgSO_4_, but none had administered it themselves.

**Conclusions:**

This study showed that knowledge regarding the aetiology of pre-eclampsia was limited. Nevertheless, their basic knowledge and skills could be strengthened to more effectively manage the hypertensive disorders of pregnancy in their communities.

**Trial registration:**

NCT01911494

**Electronic supplementary material:**

The online version of this article (doi:10.1186/s12978-016-0219-8) contains supplementary material, which is available to authorized users.

## Plain English summary

In India, pregnancy complications related to hypertension or are responsible for nearly 40 % of all maternal deaths. Most of these deaths occur in primary health facilities which frequently lack essential equipment and medication, are understaffed, and have limited access to specialist care. Community health workers are essential providers of basic maternity care in these settings. The quality of care they provide is dependent on the level of knowledge and skills they possess; however, there is limited evidence regarding their knowledge and confidence in managing pregnancy complications. This study aims to describe the current state of knowledge regarding pre-eclampsia (a complication of pregnancy characterized by high blood pressure, protein in the urine, and/or symptoms) and eclampsia (seizures in pregnancy) among community health workers (auxiliary nurse midwives, accredited social health activists, and staff nurses) in northern Karnataka State, India.

Eight focus group discussions were conducted in 2012–2013: four with staff nurses and auxiliary nurse midwives and four with accredited social health activists. In addition, twelve auxiliary nurse midwives and staff nurses completed questionnaires to explore their skills and confidence in providing obstetric care.

Community health workers provided psychological explanations for the origin of hypertension in pregnancy, such as stress, tension, and fear. Despite some common misperceptions, community health workers demonstrated a good grasp of the potential outcomes related to pregnancy complications. According to auxiliary nurse midwives and staff nurses, if hypertension was detected, they encouraged rest, decreased salt intake, iron supplementation and tetanus vaccination.

This study showed that community health workers had limited knowledge of pre-eclampsia, and their basic knowledge and skills could be strengthened to more effectively manage the hypertensive disorders of pregnancy in their communities.

## Background

India contributes significantly to the global burden of maternal mortality, as it is responsible for 17 % of all maternal deaths [1]. The current maternal mortality ratio (MMR) of India is 167 per 100,000 livebirths and 144 in Karnataka State (SRS 2010–12) [2]. The hypertensive disorders of pregnancy (HDP) are a leading causes of maternal death [3, 4], accounting for 10–15 % of all direct maternal deaths globally [3–6]. Most of these deaths occur in low- and middle-income countries (LMIC) [6]. In India, postpartum haemorrhage and the HDPs contribute to nearly 40 % of all maternal deaths [7]. The HDP are challenging because of the silent nature of the disease. Apart from being an important cause of maternal mortality, the HDPs are associated with serious maternal and neonatal morbidities.

Most maternal deaths and morbidities occur in primary health settings which lack essential resources, are understaffed, and have limited or no access to specialist care [5]. In the Indian public health system, primary health centres (PHC) serve as the first point of care. Each PHC is staffed by one doctor and three to five staff nurses, and each sub-centre is staffed by one auxiliary nurse midwife (ANM) [8, 9]. ANMs provide health services including screening, management, and referral for pregnancy and newborn complications. Since 2005, the National Rural Health Mission (NRHM) has introduced innovative strategies to accelerate progress towards improving health outcomes. These strategies include mobilization efforts by frontline workers, namely the accredited social health activists (ASHA), and numerous of initiatives to increase institutional deliveries [10]. In spite of these efforts, Karnataka State has not achieved the expected level of reduction in maternal mortality.

The community health care workers serve as the point of entry into the health system and therefore can play an important role in improving pregnancy outcomes. The proximity of these health workers to their communities is advantageous as it increases familiarity and trust. Ensuring adequate knowledge of pregnancy complications by these health workers is essential. There is limited research available regarding the knowledge and skills among community health care workers (ANM, ASHA, staff nurse) to manage pregnancy complications in India. The aim of this study was to assess the knowledge of community health workers regarding pre-eclampsia and eclampsia and how they act upon presentation of these conditions in their communities.

## Methods

**Fig. 1:**
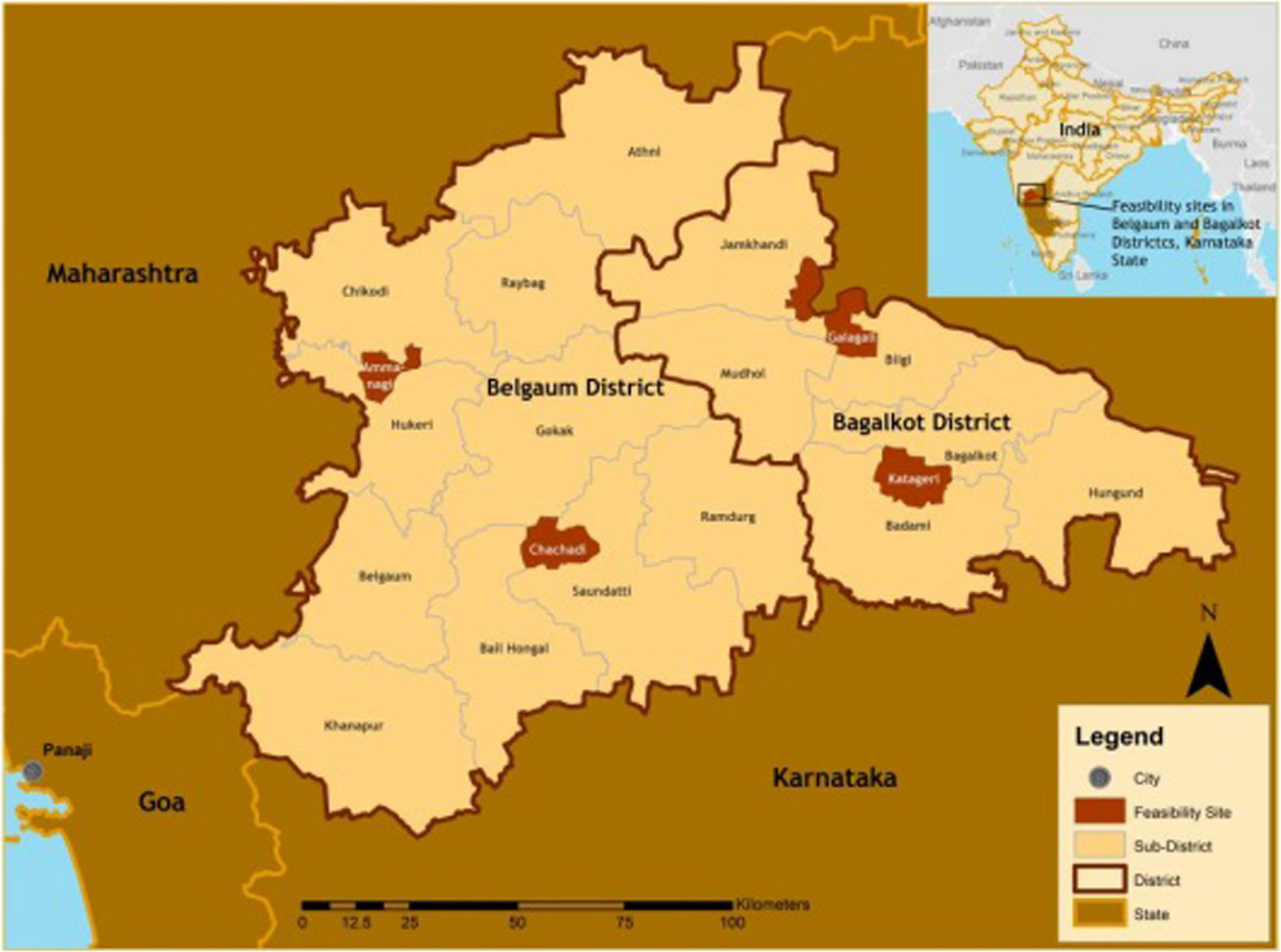
Study area, Belgaum and Bagalkot, Karnataka State, India

This study was conducted in two rural districts (Belgaum and Bagalkot) of northern Karnataka State, India (Fig. 1). Social and health indicators of Karnataka are lagging compared with other South Indian states. Health care infrastructure is often inadequate, particularly in rural areas, where there are insufficient health care providers to cover the large and dispersed population.

There are various cadres of community health workers in India, including ASHAs, ANMs and staff nurses. ASHAs act as community health activists, to counsel women on birth preparedness, the importance of safe delivery, breastfeeding, immunisation, contraception, pregnancy related ailments, sexually transmitted infections and newborn care [11, 12]. ANMs serve a larger population and their primary role is to provide basic maternal and child health care [10]. Staff nurses are trained health workers based at PHCs, they provide preventive and curative services, and are trained as skilled birth attendants [9] (Table 1). Data for this study were collected as part of a larger study aimed at assessing the feasibility of community-based identification and treatment of pre-eclampsia using community health care workers [13].

**Table 1:** Community health worker characteristics

	Staff nurse	Auxiliary nurse midwife	Accredited social health activist
Minimum education attainment	General nursing and midwifery training	10^th^ level	8^th^ level
	Bachelor of Science in nursing	Auxiliary Nurse Midwife training	
Source of payment	Government salary	Government salary	Incentives provided through national health programmes
Length of training	24–36 months	18 months	21 days
Workplace	Primary health centre	Sub-centre	Village and home
Population served	20,000–30,000	5000–6000	1000

### Focus group discussions

Focus group discussions were utilized to elucidate in-depth information and to encourage group dialogue. Local clinicians and researchers with no known association with respondents were trained to facilitate these discussions. Eight focus group discussions, four with staff nurses and ANMs and four with ASHAs were conducted at local primary health centres between January and March 2013. Data saturation was felt to be met at this time and no more focus groups were required. The discussions guides were in local language, Kannada.

All discussions were transcribed verbatim in Kannada and translated to English for analysis using NVivo 10 software. Transcripts were coded by one author (MV), after which all transcripts and themes were cross-checked by the local research team (UR, UC, GK, MB, AM, KC) to resolve any misinterpretation. Using deductive reasoning, the results were grouped into predetermined categories related to pre-eclampsia knowledge and skills. During analysis, inductive reasoning was used to incorporate new and unexpected ideas. This produced a comprehensive analysis structure to reflect the richness and variety of responses.

### Questionnaires

Questionnaires were completed by eight ANMs and four staff nurses. Some ANMs were unable to be reached as they were on leave or otherwise occupied. The questionnaire was split into two sections: one with 16 closed-ended Likert scale questions, the other with 11 open-ended questions. The purpose of this assessment was to understand the level of competence and knowledge related to pre-eclampsia amongst these ANMs and staff nurses.

This study was approved by ethics review committees at the University of British Columbia, Vancouver Canada (H12-00132) and KLE University, Belgaum India (MDC/IECHSR/2011-12). Written informed consent was obtained for all participants.

## Results

### Participants

A total of 101 community health care workers participated in this study, of which 48 were ANMs or staff nurses and the remaining 53 were ASHAs. The mean age of ANM/staff nurse was 36.5 ± 4.8 years and their years of experience ranged from 2 to 27 years. The mean age of ASHAs was 32.5 ± 0.7 years and they tended to have 5 years of experience (Table 2).

**Table 2:** Focus group discussions

#	Stakeholder	Participants	Mean age
1	Auxiliary Nurse Midwives (ANM) (*n* = 5)	8	36
	Staff nurses (*n* = 3)		
2	Auxiliary Nurse Midwives (ANM) (*n* = 6)	9	41
	Staff nurses (*n* = 3)		
3	Auxiliary Nurse Midwives (ANM) (*n* = 10)	19	39
	Staff nurses (*n* = 7)		
4	Auxiliary Nurse Midwives (ANM) (*n* = 8)	12	30
	Staff nurses (*n* = 4)		
5	Accredited Social Health Activists (ASHA) (*n* = 10)	10	32
6	Accredited Social Health Activists (ASHA) (*n* = 11)	11	--
7	Accredited Social Health Activists (ASHA) (*n* = 15)	15	--
8	Accredited Social Health Activists (ASHA) (*n* = 17)	17	33

### Danger signs of hypertension and convulsions in pregnancy

Staff nurses, ANMs and ASHAs explained that to identify pre-eclampsia, blood pressure must be measured; this is the only method of identification as symptoms cannot be used reliably to diagnose. This response showed an understanding that pre-eclampsia can develop without outward symptoms. In addition to hypertension, ANMs claimed that dizziness, swelling, visual disturbances, sweating and restlessness, were danger signs associated with pre-eclampsia. When probed regarding the signs of eclampsia, the responses included jerky movements, shaking of hands and legs, frothing from the mouth and rolling of the eyes. Both the groups provided similar findings.

### Causes of hypertension and convulsions in pregnancy

There were several explanations for the hypertensive disorders of pregnancy provided by all the three groups of community health workers. Psychological explanations of hypertension were most common: mental stress, tension, and fear. Tensions were thought to be the result of concerns related to the gender of the baby. In addition, the husband’s drinking habits or family clashes may cause tension: “*due to tension at home” [staff nurse/ANM], “repeated birth of female children, leading to mental tension” [ASHA]*. Previous caesarean deliveries were also thought to create worry, which could result in high blood pressure. There was some understanding, by health care providers, of underlying conditions that contribute to the hypertensive disorders of pregnancy. These conditions included anaemia and diabetes: “*due to anaemia she will have swelling and that will lead to BP*” *[staff nurse/ANM]*. Heredity was also believed to be associated with pre-eclampsia. Diet was explained as a contributor to the onset of pre-eclampsia: diets high in salt, oil and fried foods. Additionally, strenuous work was seen as a factor that contributes to the development of pre-eclampsia.

Similar factors were believed to be implicated in the development of eclampsia, such as tension or weakness. In addition, some health care providers stated that blood pressure was the cause of eclampsia. This explanation showed an understanding of a relationship between the two conditions. Anaemia was sometimes attributed to the incidence of eclampsia. The most common explanation of the origin of eclampsia was the lack of tetanus toxoid vaccination in pregnancy: *“If they don’t take TT [tetanus toxoid vaccine] when pregnant, they might get fits” [ANM].* Lastly, poor medical adherence was thought to play a role; the prohibitive cost and the belief that medication will result in big babies prevents some women from appropriately following treatment.

### Outcomes of hypertension and convulsions in pregnancy

Health care providers acknowledged that further consequences are likely for women suffering hypertension in pregnancy. In fact, most had a good grasp of these potential outcomes. The most serious was the possibility of death for the mother and/or baby.

The outcomes of eclampsia were thought to be associated with active seizures, disease progression, or death. Many mentioned that injuries might occur, such as broken bones, tongue bite, and aspiration: *“She may have injury due to fits episode*” [*ASHA*]. These outcomes may be to the mother, baby or both.

### Treatment of hypertension in pregnancy

Staff nurses and ANMs suggested regular blood pressure measurements in pregnancy. If hypertension was detected they advised as follows: rest in a well-lit and ventilated space, decrease salt intake, iron supplementation, and tetanus vaccination. In addition to these recommendations, staff nurses claimed to provide antihypertensive medication and in some cases MgSO_4_. ASHAs believed there was a need for regular blood pressure measurement and referral for hypertensive women. ASHAs also stressed the importance of medical adherence and the avoidance of home treatment.

Staff nurses and ANMs who reported use of antihypertensive agents, mostly commonly reported using nifidepine, though others were mentioned: atenolol, amlodipine, beta blockers, diazepam, frusemide, and MgSO_4_. Some cautioned that these medications should be given by a doctor or after consultation; while others claimed they do not provide such medication under any circumstance. ASHAs were unaware of specific antihypertensive medications, though they recognized the need for appropriate treatment by medical officers or ANMs in cases of hypertension.

### Treatment of convulsions in pregnancy

There was minimal knowledge, confidence or expertise regarding the treatment of convulsions in pregnancy. Some staff nurses stated that MgSO_4_ was the drug of choice for the treatment of eclampsia; however, few had personal experience administering it, and they were unaware of appropriate routes and dosages. Similarly, all ANMs had some knowledge of MgSO_4_, but had not administered it themselves. MgSO_4_ was perceived by some to be treatment-appropriate only in higher-level facilities by specialists. Aside from MgSO_4_, staff nurses and ANMs expressed familiarity with alternative anticonvulsants: diazepam, frusemide, and phenobarbitone. Some mentioned past use of diazepam, but explained that practice has now changed; however, one staff nurse claimed to still treat eclampsia with diazepam: “*We give injection calmpose (diazepam) and send” [staff nurse].* ASHAs did not express familiarity with MgSO_4_; some knew the name but not the indications for use.

### Clinical confidence and competence in identifying and treating hypertension and convulsions in pregnancy

**Fig. 2:**
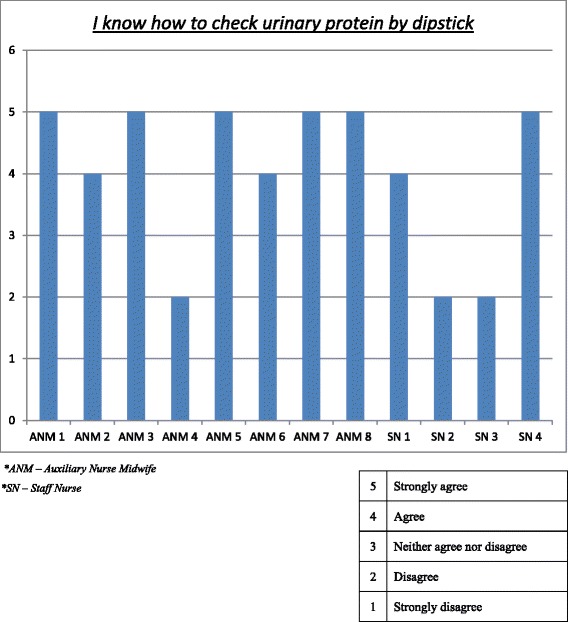
Ability to measure proteinuria by ANMs and staff nurses

It is important to identify the confidence of ANMs and staff nurses regarding identification and treatment of the HDPS; this was evaluated using a questionnaire targeting their confidence and experience. The first theme of the questionnaire probed health workers regarding preparedness, awareness, knowledge and skills related to pre-eclampsia and eclampsia. Responses were consistent between respondents with the vast majority of responses being “agree” or “strongly agree”. One exception was regarding ANM’s and staff nurse’s ability to assess proteinuria, where some respondents showed low confidence (Fig. 2).

The second theme of the questionnaire focussed on their scope of practice. Respondents claimed to monitor and refer cases of pre-eclampsia. All 12 reported to either ‘agree’ or ‘strongly agree’ to the two related statements: “*I monitor women for pre-eclampsia*” and “*I refer women for pre-eclampsia*”. The most common response regarding medication used for high blood pressure and seizures was MgSO_4_. ANMs and staff nurses also claimed to be comfortable counselling women and families to receive MgSO_4_ prior to referral (Table 3).

**Table 3:** ANM and staff nurse confidence of pre-eclampsia screening

Question	Average response	Average response by ANMs	Average response by staff nurses
		(*N* = 8)	(*N* = 4)
I know how to provide routine antenatal care to women in my catchment area	4.6	4.7	4.3
I know the danger signals of pregnancy	4.5	4.7	4.3
I know how to identify the warning signs of high blood pressure in pregnancy and postpartum	4.5	4.6	4.3
I know how to identify the warning signs of seizures in pregnancy and postpartum	4.5	4.5	4.5
I know how to identify the symptoms and signs of labour	4.6	4.7	4.5
I know how to check blood pressure	4.7	4.8	4.5
I know how to check urinary protein by dipstick	4.0	4.4	3.3
I know how to check urinary protein by boiling urine	4.0	4.4	3.3
I know how to administer vaccines to mothers and children	4.6	4.8	4.3
I know how to administer intramuscular injections to pregnant women	4.7	4.8	4.3
I know how to administer oral medications to pregnant women	4.5	4.7	4.3
I know how to directly refer pregnant women with complications	4.5	4.6	4.5

5 = strongly agree; 4 = agree; 3 = neither disagree nor agree; 2 = disagree; 1 = strongly disagree

## Discussion

The study was conducted to identify the level of knowledge held by community health care workers – staff nurses, ANM, ASHA - regarding pre-eclampsia and eclampsia and their routine management of these cases in northern Karnataka. Community health workers are essential in identifying obstetric emergencies, thus a sound knowledge of pregnancy complications is needed for early diagnosis and referral. It was observed that most community providers were aware of the link between hypertension and seizures; however, they also mentioned they can present without symptoms.

It was surprising to find that many community health providers believed missing tetanus toxoid vaccinations could lead to eclampsia; this clearly represents a misunderstanding of the underlying cause of disease. Another explanation for the hypertensive disorders of pregnancy was mental tension due to personal conflicts and the preference for a male child. In Indian culture sons are strongly preferred as they are required for the performance of funeral rites, they are expected to provide financially, and in many parts of India they command a large dowry (https://nitawriter.wordpress.com/2006/10/19/why-do-indians-prefer-sons-2/) [14]. The belief that anaemia causes hypertension was stressed by many participants; this misconception may be because of the high prevalence of anaemia in the region. The stated causes indicate significant gaps in knowledge, thus it is necessary to target improvements in the training of community health workers. Nevertheless, community health workers do seem to recognize the serious nature of pre-eclampsia and the urgent need for management and referral.

Health care providers were probed regarding their use and knowledge of antihypertensive medications, to better understand their current practice. ANMs stated that they administered antihypertensive agents when indicated; by far the most common antihypertensive in use was nifidepine. ASHAs were unaware of specific antihypertensive medications, though they recognized they should be administered by medical officers or ANMs for hypertension. It is important for skilled birth attendants to know about antihypertensive drugs, their indications, contraindications, dosage and limitations for their use.

National guidelines authorize ANMs and nurses to administer MgSO_4_ to women suffering from eclampsia; however, the majority of ANMs claimed not to have administered MgSO_4_ themselves. There are differences in the professional qualification, job responsibilities, and practical experience of the staff nurses and ANMs; however, practice guidelines are combined and so do not differentiate these responsibilities creating confusion.

Participants stated that they can administer intramuscular injections and oral medications in pregnancy, this self-reported skill can very well be utilized in the management of HDPs; however, it is not clear weather this competence translates to the use of MgSO_4_. This lack of experience with MgSO_4_ clearly indicates a gap in knowledge and the implementation of guidelines. Even though community health workers understood that pre-eclampsia and eclampsia can lead to severe outcomes, their knowledge of treatments was inadequate. The majority of ANMs misclassified MgSO_4_ as an antihypertensive agent and nifedipine as an anticonvulsant for eclampsia. These misunderstandings could lead to adverse effects; in addition, some providers reported use of diazepam which is known to be dangerous in pregnancy. The misclassification of antihypertensives and anticonvulsants by ANMs highlights knowledge gaps that must be addressed.

The large sample size and mixed methods used for this study strengthens the findings. Due to the qualitative nature of this study, the results are not generalizable to other groups or regions. The findings of this study cannot be deemed representative of all community health workers in India; nevertheless they provide insight into the knowledge gaps and common misconceptions related to the hypertensive disorders of pregnancy. Focus group discussions provided detailed insight into the experience and beliefs of community-based health workers; however, the ideas given by a small number of participants may have influenced others. Some focus groups included many participants which may have decreased the facilitator’s ability to engage with the whole group; however, this approach allowed participation from a large group of respondents and many potential points of view.

### Implications

Due to the identified knowledge gaps and common misconceptions there is a need to update training and regular assessment of community health worker competence and confidence regarding the identification and treatment of the hypertensive disorders of pregnancy. The national guidelines that authorize ANMs to administer MgSO_4_ and antihypertensives need to be broadly disseminated so ANMs are fully aware of what their scope of practice includes. Professional practice guidelines that are combined for ANMs and staff nurses should be separated to avoid confusion in their scope of practice. These changes are required at the regulatory and systems level so that ANMs and staff nurses have full clarity and support in provision of appropriate and safe patient care during pregnancy and childbirth.

## Conclusion

Community health care workers are the link between the health care system and the community. The knowledge and practices of these providers can have a profound effect on maternal health outcomes. This study demonstrated that community health workers had limited knowledge of aetiology of pre-eclampsia, as hypertension was most commonly attributed to stress, and eclampsia was attributed to a lack of tetanus vaccinations, anaemia, and poor medical adherence. More importantly, most of the health workers acknowledged the serious nature of the disease and were aware of appropriate medications, though not the route and dosage. It is important to build upon the knowledge gaps identified to most effectively improve maternal and perinatal health outcomes.

## Additional file

Additional file 1: Reviewer reports. (PDF 214 kb)

## Abbreviations

ANM: Auxiliary nurse midwife
ASHA: Accredited social health activist
HDP: Hypertensive disorders of pregnancy
LMIC: Low- and middle-income countries
MDG: Millennium development goals
MgSO_4_: Magnesium sulphate
MMR: Maternal mortality ratio
NRHM: National Rural Health Mission
PHC: Primary health centre
