# Supplementary material for: Community health worker knowledge and management of pre-eclampsia in rural Karnataka State, India
Source: Reprod Health. 2016 Sep 30;13(Suppl 2):113. doi: 10.1186/s12978-016-0219-8 (PMC5056468; doi:10.1186/s12978-016-0219-8)
Supplement: Additional file 1: — Reviewer reports. (PDF 214 kb) [file 12978_2016_219_MOESM1_ESM.pdf]

Referee's comments to the authors– this sheet WILL be seen by the author(s) and published with the article

Title	Community health worker knowledge and management of pre-eclampsia in India
Author(s)	Umesh Ramadurg, Marianne Vidler, Umesh Charanthimath, Geetanjali Katageri, Mrutyunjaya Bellad, Ashalata Mallapur, Shivaprasad Goudar, Shashidhar Bannale, Chandrashekhar Karadiguddi, Diane Sawchuck, Rahat Qureshi, Peter von Dadelszen, Richard Derman for the Community Level Interventions for Pre-eclampsia (CLIP) India Feasibility Working Group
Referee's name	Shuchita Mundle

When assessing the work, please consider the following points, where applicable:

1. Is the question posed by the authors new and well defined?
2. Are the methods appropriate and well described, and are sufficient details provided to replicate the work?
3. Are the data sound and well controlled?
4. Does the manuscript adhere to the relevant standards for reporting and data deposition?
5. Are the discussion and conclusions well balanced and adequately supported by the data?
6. Do the title and abstract accurately convey what has been found?
7. Is the writing acceptable?

Please make your report as constructive and detailed as possible in your comments so that authors have the opportunity to overcome any serious deficiencies that you find and please also divide your comments into the following categories:

- Major Compulsory Revisions (which the author must respond to before a decision on publication can be reached)
- Minor Essential Revisions (such as missing labels on figures, or the wrong use of a term, which the author can be trusted to correct)
- Discretionary Revisions (which are recommendations for improvement but which the author can choose to ignore)

Where possible please supply references to substantiate your comments.

When referring to the manuscript please provide specific page and paragraph citations where appropriate.

General comments: The topic of the paper is very relevant and the findings would be important to implement interventions.

Major compulsory revisions:
Methods: Focus group discussions (FGDs): Health care provision at the community level is a responsibility of the Nurses and ANM, both cadres being more qualified and trained than the ASHA workers. The percentage participation of AHSA workers is more than 50% in the FGDs. The results from these discussions cannot be combined as representative of the community health workers.

The number of participants in FGD's is not ideal (Table 2,row 3,7 and 8).
There is no break up of the number of nurses and ANM in the focus groups, table 2.

Interviews: The details of sampling are not given. The sample size for interviews with community health workers is not adequate to make conclusions about the two geographic regions.

Table 3 shows combined responses from ANM and staff nurses, discussion points out to the differences in training for these two cadres. (#300,301,302)

Minor essential revisions:
#105-106: No reference for this statement, given reference is wrongly numbered.

#168-170: 'majority of the participants had undergone skilled birth attendant training': 57% of the participants had training, which in shown in Table 2, and not Table 3.

Chart on the last page (unnumbered) is incomplete.

Referee's comments to the authors– this sheet WILL be seen by the author(s) and published with the article

Title	Community health worker knowledge and management of pre-eclampsia in India
Author(s)	Umesh Ramadurg, Marianne Vidler, Umesh Charanthimath, Geetanjali Katageri, Mrutyunjaya Bellad, Ashalata Mallapur, Shivaprasad Goudar, Shashidhar Bannale, Chandrashekhar Karadiguddi, Diane Sawchuck, Rahat Qureshi, Peter von Dadelszen, Richard Derman for the Community Level Interventions for Pre-eclampsia (CLIP) India Feasibility Working Group
Referee's name	Chandrika N Wijeyaratne

When assessing the work, please consider the following points, where applicable:

1. Is the question posed by the authors new and well defined?
2. Are the methods appropriate and well described, and are sufficient details provided to replicate the work?
3. Are the data sound and well controlled?
4. Does the manuscript adhere to the relevant standards for reporting and data deposition?
5. Are the discussion and conclusions well balanced and adequately supported by the data?
6. Do the title and abstract accurately convey what has been found?
7. Is the writing acceptable?

Please make your report as constructive and detailed as possible in your comments so that authors have the opportunity to overcome any serious deficiencies that you find and please also divide your comments into the following categories:

- Major Compulsory Revisions (which the author must respond to before a decision on publication can be reached)
- Minor Essential Revisions (such as missing labels on figures, or the wrong use of a term, which the author can be trusted to correct)
- Discretionary Revisions (which are recommendations for improvement but which the author can choose to ignore)

Where possible please supply references to substantiate your comments.

When referring to the manuscript please provide specific page and paragraph citations where appropriate.

General comments: The purpose of this study is ill matched with the methodology adopted, particularly that he sampling technique adopted does not represent the population of India – as stated in the title

Major compulsory revisions: The high mortality from pre eclampsia/eclampsia cannot solely be attributed to a poorly directed filed level staff. The multiple stakeholders in safe motherhood needs to be considered – not merely voluntary social health workers who no doubt will have their own opinions on the evolution of life threatening maternal disease that has multiple socio-cultural perspectives. Hence reporting and concluding that the health care providers/facilitators at grassroots level attributing the ack of immunization against tetanus to be the cause of maternal hypertensive disease is flawed scientifically. This is particularly so in qualitative research where a 'fish bone approach' to finding the root causes is required. A more systematic approach to arriving at conclusions that would help in identifying and thereby testing more appropriate interventions should be the main basis of such research reports.

Supplement Editor comments
Both reviewers raised serious concerns about your article one of them suggesting rejection.
The major concern of the reviewer suggesting rejection is based on the lack of representativeness of you study avoiding the mention of the entire country in the title and throughout the text.
The editorial decision is to give you an opportunity to address these major concerns and once receiving your reply to make a decision about.
